# The Effect of Natural Hazard Damage on Manufacturing Value Added and the Impact of Spatiotemporal Data Variations on the Results

**DOI:** 10.1007/s13753-022-00438-x

**Published:** 2022-09-16

**Authors:** Douglas Thomas, Jennifer Helgeson

**Affiliations:** National Institute of Standards and Technology, Gaithersburg, MD 20899 USA

**Keywords:** Manufacturing, Natural hazards, Spatiotemporal impacts, United States, Value added

## Abstract

This study examined the effect of natural hazards on manufacturing industry value added and the sensitivity of the results from changes to spatiotemporal resolution of the data. We measured the negative effects of hazards, rather than the net effect. Three models were developed with varying spatiotemporal units for the continental United States: annual/county units; annual/state units; and quarterly/state units. Three simulations were run using each model to estimate the negative effect of damage from all natural hazards on value added across spatiotemporal scales. Finally, an investment analysis was conducted to examine the return from public investments in hazard resilience. The results do not demonstrate that, locally, natural hazards reduce value added. However, the evidence suggests that natural hazards in the upstream supply chain have statistically significant impact when modeled at the annual/county scale and at the quarterly/state scale. Neither local nor supply chain hazards have a statistically significant effect when modeled at the annual/state scale, suggesting that broader spatiotemporal units may obscure the true downstream effects of natural hazards. The investment analysis, utilizing model results, suggests that an investment of USD 100 billion or less is economical if it results in a reduction in losses of 10% or more.

## Introduction

In 2020–2021 there were 42 weather/climate disaster events with losses exceeding USD 1 billion each affecting the United States (NCEI 2021). These natural hazards took place in tandem with the global coronavirus disease 2019 (COVID-19) pandemic to create compounded risks and resulted in complex event impacts for many businesses, especially small- and medium-sized enterprises (Helgeson et al. 2021). Indirect and ancillary net costs to supply chains during and following natural hazards are frequently left unaccounted for in the overall impact assessment (Rose 2009; Oh et al. 2012). This is especially true for hazards that strike at locations geographically separate from where financial impacts may be observed. “Propagation of impact throughout the supply chain and the connection between the local disaster impact and impacts felt elsewhere are not often part of the disaster loss calculation” (Thomas and Helgeson 2021, p. 1). Despite numerous supply-chain upheavals caused by disasters in the last two decades—including the volcanic eruption in Iceland, Japanese earthquake and tsunami, Thailand floods, the immense 2017 hurricane season in the United States, and increasing wildfires worldwide—there continues to be minimal supply chain emergency planning (UNDRR 2019).

There has been research on indirect losses via propagation through supply chains to regions not directly hit by a natural hazard; a review of relevant research is available in Thomas and Helgeson (2021). Yet, much of the literature to date remains focused at the enterprise-level or towards global supply chain trends, as opposed to examining inter-regional and indirect impacts. In their review of 20 years of supply chain disruption research, Katsaliaki et al. (2021) find that supply chain disruption has been emphasized in the literature; however, notable gaps remain. Among these gaps is a deeper understanding of losses across locations and time periods post-event, which we refer to as the spatiotemporal dimension.

This article expands upon findings in Thomas and Helgeson (2021) to look at US supply chain data trends to assess: (1) How hazard damage occurring locally reduces manufacturing value added in subsequent years/quarters; and (2) How the ripple effects of hazard damage occurring in the supply chain reduces manufacturing value added in subsequent time periods. We estimate the negative effect of damage from all natural hazards on value added across spatiotemporal scales. Finally, an investment analysis was conducted to examine the possible return-on-investment towards supply chain hazard resilience. The results do not demonstrate that, locally, natural hazards reduce value added.

This article is organized as follows. Section 2 provides background and additional context to the problem statement and associated research hypotheses. We then provide a description of the data in Sect. 3 and methods used in our analysis in Sect. 4. Results and discussion follow in Sect. 5; and future research directions conclude in Sect. 6.

## Background and Context

Current global supply chain networks are vulnerable to a variety of risks; these have been broadly documented in the literature (Tang 2006; Rose 2009). In this article we conceptualize supply chain disruption as an unintended, untoward situation, which leads to supply chain risk, following from the definition used by Wagner and Bode (2006). Supply chain disruptions can materialize from supply-side and/or demand-side risks and both these types of risk can be exacerbated by natural hazards and disasters (Thomas and Helgeson 2021).

There is a wide literature on supply chain disruption risks; for a review see Katsaliakiet al. (2021); some assessments take into account spatiotemporal ripple effects post-disasters, but do so in limited ways (Tang 2006; Li 2014; DHS 2019). Supply chain disruption risks generally fall into three broad categories: (1) organizational; (2) supply chain-related; or (3) environmental. These risks in turn propagate and are exacerbated through network factors (that is, characteristics of the supply chain) and external factors (that is, characteristics beyond the direct influence of the supply chain network).

Natural hazards and disasters are an increasingly frequent cause of supply chain disruption. The direct impact on an industry can be significant, especially during lengthy recovery periods that are riddled with disruptions to critical lifeline services (for example, utility availability) (DHS 2019; McAuliffe and Khadria 2019). However, assessment of indirect and secondary damages and disruptions—which we know are of high magnitude anecdotally—is difficult due to intangibility and distributed impacts that are difficult to rank and quantify (Henriet et al. 2012). As natural hazards are expected to increase in intensity and frequency (IPCC 2021), it becomes increasingly important to assess these indirect and ancillary losses, especially through ripple effects along the supply chain. The ripple effect “describes the impact of a disruption propagation on supply chain performance and disruption-based scope of changes in supply chain structural design and planning parameters” (Dolgui et al. 2018, p. 1).

The intensity of the ripple effect caused by natural hazards varies with prevailing trends in supply chain management (SCM). On the one hand, supplier consolidation by firms lowers transaction costs and enhances partnerships in time of business-as-usual. Furthermore, there are trends towards production firm agglomeration, which lends itself to knowledge spillover, labor market pooling, and lower shipping costs (Dong 2020). Yet prevailing SCM practices may limit firms’ ability to cope with supply and demand shocks and market volatility generated by disaster events (Alesch et al. 2001; Wedawatta and Ingirige 2012). Increasingly SCM operational choices are being made in an attempt to lessen vulnerability to shocks (McKinsey Global Institute 2020; Bui et al. 2021).

Data on the economic impacts of disaster events that inherently consider supply chain, but do not explicitly assess impacts propagated via supply chains, present mixed evidence on whether economic growth is negatively impacted (Benson and Clay 2004; Cavallo and Noy 2009; Koks and Thissen 2016; Mohan et al. 2019). Loayza et al. (2012) found that moderate disasters can lead to moderate (localized) growth in some sectors, especially in the short term, while more severe disasters do not. This may arise from the opportunity for accelerated updates to (local) capital through near-term increased demand in some sectors (Hallegatte and Dumas 2009); however, there is the potential for permanent deviations from previous growth trajectories when the system is not resilient (Skidmore and Toya 2002).

Indirect and ancillary losses can be triggered across the region directly impacted by a natural hazard through secondary impacts on the region’s production and trade networks. Shughrue et al. (2020) found that these types of impacts account for up to 75% of the total regional damage. Even minor natural hazards can have ancillary impacts on households through their position as both consumers and suppliers of labor over periods well beyond a year after an event (Watson et al. 2020). Additionally, natural hazards may impact spatially disparate parts of the supply chain via unanticipated demand, rush orders, shortage in supply, company buyouts, delivery coordination, and sourcing constraints (Scheibe and Blackhurst 2018).

As described in Thomas and Helgeson (2021) and summarized here, there are at least three nontrivial reasons that downstream ripple effects across regions and over time should be considered: (1) Many data observations are needed to reveal the risks of natural hazards that are infrequent, though their frequency and strength are increasing; (2) Without a thorough understanding of the costs and losses from natural hazards, there may be underinvestment in risk mitigation research that extends beyond typical geographic boundaries; and (3) An understanding of the net potential losses across the economy helps make the case for risk mitigation actions to be incentivized for individual entities along the supply chain, which may otherwise not take action.

In the current article, we build upon our previous analysis (Thomas and Helgeson 2021) that shows that the compound effect of hazards through the supply chain exceeds that of the local hazard (that is, direct impacts), which in turn creates the potential for incentive misalignment between the establishment investing in hazard impact mitigation and those firms that benefit the greatest from mitigation. In the present article we address the need to estimate the spatiotemporal distribution of ripple effects to allow for a better understanding of the need for resilience planning in locations that may not be traditionally considered and have direct implications in determining the level of public investment in resilience. Additionally, we examine the effect of spatiotemporal resolution on the results of the analysis and use the models to conduct an investment analysis of public investment in hazard resilience.

## Data

This study used three primary datasets, including data from the U.S. Bureau of Economic Analysis, U.S. Department of Transportation, and Arizona State University. Data on value added for total manufacturing, durable goods manufacturing, and nondurable goods manufacturing were obtained from the U.S. Bureau of Economic Analysis. These data include county (Bureau of Economic Analysis 2020) and state level (Bureau of Economic Analysis 2021) real GDP in chained 2012 dollars by industry, which measure the value of the nation’s output. Industries are categorized by the North American Industry Classification System (NAICS).

**Fig. 1:**
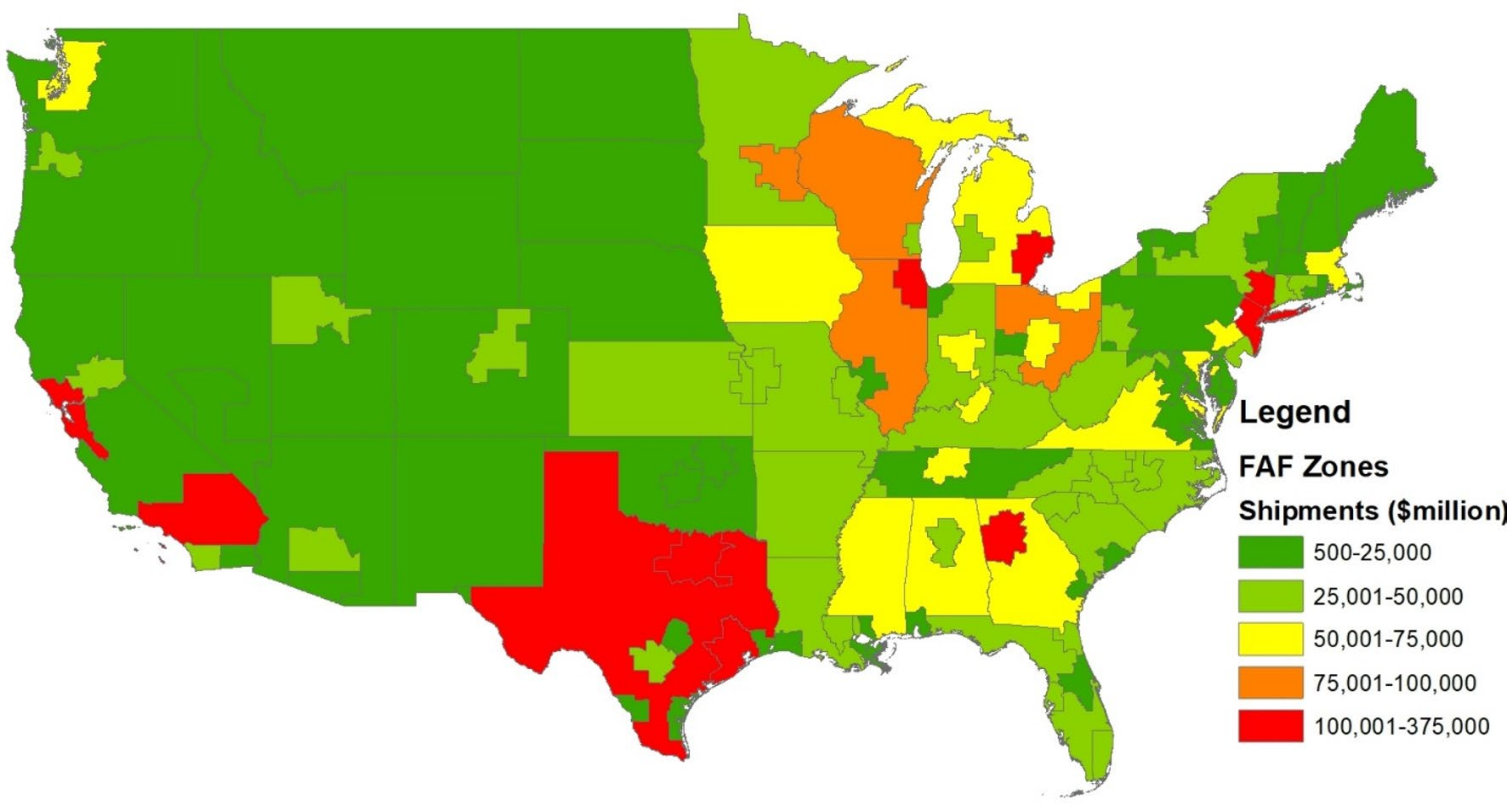
Shipments by Freight Analysis Framework (FAF) origin, lower 48 states of the United States (2016). *Data source* U.S. Department of Transportation (2018)

Data from the Freight Analysis Framework (FAF; Department of Transportation 2018) that tracks shipments within the United States were used to examine disruptions in the supply chain. The FAF data are categorized by the Standard Classification of Transported Goods and include shipments between 122 zones (see Fig. 1) covering the entire United States for the period 2012 through 2016. For this analysis, 13 categories of commodities were used (see Table 1). These categories were selected to represent intermediate goods along with goods that likely have lower levels of substitutability.

**Table 1:** Commodities in analysis

20 Basic Chemicals
23 Chemical Products
24 Plastics and Rubber
25 Logs and Other Wood in the Rough
26 Wood Products
30 Textiles, Leather, and Articles of Textiles or Leather
31 Nonmetallic Mineral Products
32 Base Metal in Primary or Semi-finished Forms and in Finished Basic Shapes
33 Articles of Base Metal
34 Machinery
35 Electronic and Other Electrical Equipment and Components, and Office Equipment
36 Motorized and Other Vehicles (including parts)
37 Transport Equipment (Other)

Hazard count and damage data from the Spatial Hazard Events and Losses Database for the United States (SHELDUS) accessed through Arizona State University (2018) were used in the analysis. The SHELDUS™ includes natural hazards such as hurricanes, thunderstorms, and floods along with perils such as flash floods and heavy rainfall that may be considered “stressors.” The data used include hazards and perils, which is defined in the database as including (but not limited to): earthquake, flooding, fog, hail, heat event, hurricane, tropical storm, landslide, lightning, thunderstorm, tornado, tsunami, volcano, wildfire, wind event, and winter weather. Note that there may be some imprecision in damage level estimates as damages are often reported in round numbers such as USD 50,000 or USD 75,000, suggesting there is some level of detail that is missing. This imprecision can diminish statistical significance, making our estimates more conservative.

## Methods

Using a series of regression analyses this study examined two main hypotheses:

### Hypothesis 1

Hazard damage occurring locally reduces manufacturing value added in subsequent years/quarters.

### Hypothesis 2

Hazard damage occurring in the upstream supply chain reduces manufacturing value added in subsequent years/quarters.

These hypotheses are examined by way of three models that vary by the spatial units and time units employed. The first model examines the effect of hazard damage at the county level using annual data. The second model is at the state level also using annual data and the third model is at the state level but uses quarterly data. The models can be characterized as follows. Model 1: refined spatial scale with broad temporal scale; Model 2: broad spatial scale and broad temporal scale; Model 3: broad spatial scale with refined temporal scale.

Comparing the results of these three models also reveals the effect of examining the impacts of hazards at different spatiotemporal units. Thus, a third hypothesis, regarding spatiotemporal scale, is examined:

### Hypothesis 3

Given that natural hazards tend to be local in nature and given that they tend to be short duration aperiodic events, broader spatiotemporal units will tend to obscure the effect on GDP, resulting in an absence of statistical significance for the effects of natural hazards.

The idea behind this hypothesis is that the effects of natural hazards might become lost in the noise at larger scales. Moreover, if Hypothesis 3 is true, we should expect that Model 2, which has both broad spatial scale and broad temporal scale, to have an absence of statistical significance for the hazard damage variables both locally and in the supply chain.

### Regression

This study used the models presented by Thomas and Helgeson (2021), which employ a Cobb–Douglas production function. Research from Shughrue et al. (2020) and Koks and Thissen (2016) confirmed that damages grow nonlinearly, which suggests that nonlinear models such as the Cobb–Douglas may more accurately measure the effects of hazards. The effects of damages are multiplicative and exponential, as discussed in Thomas and Helgeson (2021). The Cobb–Douglas model was selected because it both captures the multiplicative/exponential relationship and facilitates using lagged dependent variables in place of capital, labor, and technology components. However, since the models in this study do not strictly follow a Cobb–Douglas production function, it might also simply be considered a logarithmic transformation (Kennedy 2003).

The models used in this study contain 10 variable groupings plus two individual variables: (1) lagged dependent variables; (2) local hazard damage; (3) local hazard count; (4) interaction of local hazard damage and the dependent variable; (5) interaction of hazard count and the dependent variable; (6) hazard damage supply chain variable; (7) hazard count in the supply chain; (8) zero local damage indicator; (9) zero count indicator; (10) indicator for each quarter (only for the quarterly model); (11) indicator for 2012 and earlier; (12) indicator for negative GDP growth nationally. Below is a discussion on why each variable is included.

The Cobb–Douglas production function uses capital, research and development, labor, and technological progress. To control for these items, our models use lagged values of the dependent variable, manufacturing GDP. Because GDP follows seasonal patterns, we use indicator variables for the second, third, and fourth quarters. Additionally, to prevent economic downturns from creating spurious correlations, we include an indicator variable for when national GDP declines.

The first group of variables that we are interested in is local hazard damage, measured in US dollars, and we examine the effect the damage has for up to 2 years. Supply chains can be years long, as Thomas and Kandaswamy (2015) demonstrate, which is why we examine the impact for up to 2 years. Hazard events can have both positive and negative effects on GDP through local hazards. For instance, there is a positive economic impact when companies and consumers increase spending to replace damaged property, but there is a negative impact when hazards damage infrastructure needed to facilitate production activities. A count variable is intended to capture any positive effects of a hazard (for example, expenditures on repairs or public aid) while the damage variable is intended to capture negative effects. However, it is not possible to completely separate positive and negative effects; thus, any measured negative effects are potentially a lower bound, as some positive effects may be countering the negative ones. Note that we are primarily interested in the negative effects. Although there may be some establishments that benefit from a hazard, that is little consolation for those that did not benefit and whose business is disrupted or damaged. Generally, the businesses that benefit are those that receive funds or an increase in purchases to address or replace losses. Those that suffer are the ones that have losses to replace and those who have disruptions in their supply chain. Gross domestic product represents the economic activity of all firms in a geographic location over a specified period of time, regardless of whether they benefited or suffered from a hazard. We are examining the GDP in the location of a hazard and that of those that receive a significant amount of the supplies from the location of the hazard (that is, those in the downstream supply chain). Downstream losses suggest the potential for a misalignment of incentives, as the firm that experiences the hazard does not bear all the losses, resulting in an underinvestment in resilience. Each lag of the count and damage variable is interacted with manufacturing GDP to account for the scale of damage relative to the level of production at a given location.

A variable representing hazard damage in the supply chain was developed by taking the damage at each of the FAF supply chain zones in the top 20% for a location, multiplying the damage by the proportion of domestic shipments, and summing the product. For instance, consider Frederick County in the State of Maryland. The supply chain variable for hazard damage (that is, ${\mathrm{SUPCHN}}_{t-m,x}$ described below, where $x$ is Frederick County at time $t-m$ where $t$ is in years) for this location is the amount shipped to that FAF zone from the largest supplier (excluding self-supply) divided by the total shipped to the region from all US locations (including FAF region self-supply). This proportion is multiplied by total hazard damage in the FAF supplier zone. This calculation is made for each of the top 20% of FAF zones for Frederick (that is, top 25 locations) and summed together. Moreover, this variable represents damage occurring in the majority of a supply chain for a particular location (for example, Frederick County) each weighted by the amount of the supply chain it represents for that location. The top 20% of the FAF zones is used as this typically accounts for 80% of the supply chain. For the state level models, it is the top 10 supplier locations and for the county analysis it is the top 25 supplier locations. Similar to the local hazard damage variable and count variable, there is a supply chain variable for the total number of hazards. This variable is meant to capture any positive impacts from natural hazards.

This analysis uses value added data from 2001 through 2016; however, data on the supply chain from the Department of Transportation is available from 2012. Therefore, 2012 is used to measure shipments from 2001 through 2012 and an indicator variable is included in the model for these years. That is, the selection of supply chain locations and weighting of the hazard damage in the supply chain do not vary for years 2001 through 2012. This is not likely to cause an issue as shipments change slowly over time; thus, the same or similar supply chain locations would likely have been used with similar levels of shipments used to weight hazard damage. The primary variation is likely to be the hazard damage itself, which is the variable that we are examining.

The models examine GDP at the county and state levels. The regression equation in log terms for the models are represented as:

$$\mathrm{ln}\left({\mathrm{GDP}}_{t,x}\right)=\sum_{m=1}^{n}{\beta }_{m}\mathrm{ln}\left({\mathrm{GDP}}_{t-m,x}\right)+\sum_{m=1}^{n}{\beta }_{m+n}\mathrm{ln}\left({\mathrm{HZRD}}_{\mathrm{DMG},t-m,x}\right)+\sum_{m=1}^{n}{\beta }_{m+2n}\mathrm{ln}\left({\mathrm{HZRD}}_{\mathrm{CNT},t-m,x}\right)+{\mathrm{INTRCT}}_{1}+{\mathrm{INTRCT}}_{2}+\sum_{m=1}^{n}{\beta }_{m+5n}{\mathrm{SUPCHN}}_{t-m,x}+\sum_{m=1}^{n}{\beta }_{m+6n}{\mathrm{HZRD}}_{\mathrm{SC},t-m,x}+\sum_{m=1}^{n}{\beta }_{m+7n}\mathrm{ln}\left({\mathrm{ZERO}}_{\mathrm{DMG},t-m,x}\right)+\sum_{m=1}^{n}{\beta }_{m+8n}\mathrm{ln}\left({\mathrm{ZERO}}_{\mathrm{CNT},t-m,x}\right)+\sum_{p=2}^{4}{\beta }_{p+9n}{Q}_{p}+{\beta }_{4+9n}\mathrm{YR}+{\beta }_{5+9n}{\mathrm{GDPNEG}}_{x}+{\beta }_{6+9n}+\mathcal{E},$$

where

$${\mathrm{INTRCT}}_{1}=\sum_{m=1}^{n}{\beta }_{m+3n}\left[\mathrm{ln}\left({\mathrm{HZRD}}_{\mathrm{DMG},t-m,x}\right)\times \mathrm{ln}\left({\mathrm{GDP}}_{t-m,x}\right)\right],$$

$${\mathrm{INTRCT}}_{2}=\sum_{m=1}^{n}{\beta }_{m+4n}\left[\mathrm{ln}\left({\mathrm{HZRD}}_{\mathrm{CNT},t-m,x}\right)\times \mathrm{ln}\left({\mathrm{GDP}}_{t-m,x}\right)\right],$$

$${\mathrm{SUPCHN}}_{t-m,x}=\sum_{z=1}^{25}\frac{{\mathrm{SC}}_{t-m,\mathrm{Top}-z}}{\sum_{\mathrm{i}=1}^{122}{\mathrm{SC}}_{t-m,i}}{\mathrm{HZRD}}_{\mathrm{DMG},t-m,\mathrm{Top}-z},$$

${\mathrm{GDP}}_{t,x}$ = GDP for all manufacturing for time $t$ by geography $x$, where time is in years for Model 1 and Model 2 or quarters for Model 3. The variable $x$ is either a county for Model 1 or a state for Model 2 and Model 3;

$n$ = Number of time units for two years. For annual data, $n$ equals 2 and for quarterly data, $n$ equals 8;

${\mathrm{HZRD}}_{\mathrm{DMG},t-m,x}$ = The total damage in geography $x$ (county or state) caused by all hazards and perils listed in the SHELDUS database lagged by $m$ number of time units (years or quarters);

${\mathrm{HZRD}}_{\mathrm{CNT},t-m,x}$ = The total number of hazards and perils from SHELDUS in geography $x$ (county or state) listed in the SHELDUS database lagged by $m$ time units (quarters or years);

${\mathrm{HZRD}}_{\mathrm{SC},t-m,x}$= The total number of hazards and perils listed in SHELDUS for all counties in the top 20% of supply chain zones for geography $x$ (county or state) at time $t-m$ in years or quarters;

$\mathrm{YR}$ = Indicator variable for 2011 and earlier where $\mathrm{ln}\left(\mathrm{YR}\right)$ equals 1 when the observation year is less than 2012;

${\mathrm{SC}}_{t-m,\mathrm{Top}-z}$ = The value of selected shipments shown in Table 1 supplied to location *x* from the *z*th largest supplier where *z* is between 1 and 25;

${\mathrm{SC}}_{t-m,i}$ = The value of selected shipment types shown in Table 1 supplied to location *x* from location $i$ where $i$ is 1 through 122 of the FAF regions;

${\mathrm{ZERO}}_{\mathrm{CNT},t-m,x}$ = Indicator variable for zero hazard incidents locally for time $t-m$ in county *x* where $\mathrm{ln}\left({\mathrm{ZERO}}_{\mathrm{CNT},t-m,x}\right)$ equals 1 when there are zero hazard incidents;

${\mathrm{ZERO}}_{\mathrm{DMG},t-m,x}$ = Indicator variable for zero hazard damage locally for time $t-m$ in county *x* where $\mathrm{ln}\left({\mathrm{ZERO}}_{\mathrm{DMG},t-m,x}\right)$ equals 1 when there are zero hazard incidents;

${\mathrm{GDPNEG}}_{x}$ = Indicator for national negative growth in GDP in time period $x$;

${Q}_{p}$ = Indicator variables for quarter $p$, where $p$ is quarter two, three, or four for quarterly time units. Note that these variables are absent in the annual models;

$$\mathcal{E}$$ = Error term;

${\beta }_{y}$ = Parameter set to be estimated where *y* is parameter 1 to the total number of parameters.

To further substantiate the results, an alternative model set was created that removes the hazard damage variables (${\mathrm{HZRD}}_{\mathrm{DMG},t-m,x}$ and ${\mathrm{SUPCHN}}_{t-m,x}$) that were not statistically significant. The associated count variables (${\mathrm{HZRD}}_{\mathrm{CNT},t-m,x}$) and zero hazard indicators (${\mathrm{ZERO}}_{\mathrm{CNT},t-m,x}$) were also removed.

Multiple versions of the Breusch–Pagan and Cook–Weisberg test for heteroskedasticity (Stata 2013a) indicated the presence of heteroskedasticity. This issue was addressed using a fixed-effects model using a GLS estimator (producing a matrix-weighted average of the between and within results) (Stata 2013b), which has been shown to provide robust estimates for data with this issue (Hoechle 2007).

### Simulation

Using the regression model and results, a simulation was conducted to estimate the total negative effect that hazards occurring locally and in the supply chain have on manufacturing value added, similar to that conducted in Thomas and Helgeson (2021). We focus on the negative effects, as we are interested in measuring the losses that occur prior to recovery and excluding those establishments that might benefit from a hazard such as when purchases increase to replace or repair damage. Some establishments might benefit from a hazard; however, that is little consolation for those who are negatively affected. We ran a simulation predicting hazard damage over the study period using the estimated parameters. The estimate of manufacturing value added was then compared to two simulations where: (1) no damage occurred locally; and (2) no damage occurred in the supply chain. Zero damage locally was estimated by setting hazard damage (${\mathrm{HZRD}}_{\mathrm{DMG},t-m,x}$) equals to 1 and the indicator variable for zero damage (${\mathrm{ZERO}}_{\mathrm{DMG},t-m,x}$) set so that $\mathrm{ln}\left({\mathrm{ZERO}}_{\mathrm{DMG},t-m,x}\right)$ equals 1. This was calculated only for those hazard damage variables that were statistically significant. The percent change in manufacturing value added was calculated as:

$${\mathrm{PC}}_{\mathrm{LOC}}=\frac{\sum_{x=1}^{n}\left({\mathrm{DEP}}_{\mathrm{LOC},\mathrm{DMG},x}-{\mathrm{DEP}}_{\mathrm{LOC},\mathrm{NO}-\mathrm{DMG},x}\right)}{\sum_{x=1}^{n}{\mathrm{DEP}}_{\mathrm{LOC},\mathrm{NO}-\mathrm{DMG},x}}\times 100,$$

where

${\mathrm{PC}}_{\mathrm{LOC}}$ = Percent change in manufacturing value added resulting from no hazard damage locally;

${\mathrm{DEP}}_{\mathrm{LOC},\mathrm{DMG},x}$ = Estimate of manufacturing value added in location *x* estimated with local hazard damage;

${\mathrm{DEP}}_{\mathrm{LOC},\mathrm{NO}-\mathrm{DMG},x}$ = Estimate of manufacturing value added in county *x* estimated with no local hazard damage.

A similar examination was made with and without damage in the supply chain, where manufacturing value added is estimated with ${\mathrm{SUPCHN}}_{z}$ equaling 1 or USD 1 in damage. The percent change was then calculated:

$${\mathrm{PC}}_{\mathrm{SUPCHN}}=\frac{\sum_{x=1}^{n}\left({\mathrm{DEP}}_{\mathrm{SUPCHN},\mathrm{DMG},x}-{\mathrm{DEP}}_{\mathrm{SUPCHN},\mathrm{NO}-\mathrm{DMG},x}\right)}{\sum_{x=1}^{n}{\mathrm{DEP}}_{\mathrm{SUPCHN},\mathrm{NO}-\mathrm{DMG},x}}\times 100,$$

where

${\mathrm{PC}}_{\mathrm{SUPCHN}}$ = Percent change in manufacturing value added due to hazard damage in the supply chain;

${\mathrm{DEP}}_{\mathrm{SUPCHN},\mathrm{DMG},x}$ = Estimate of manufacturing value added in county *x* estimated with hazard damage in the supply chain;

${\mathrm{DEP}}_{\mathrm{SUPCHN},\mathrm{NO}-\mathrm{DMG},x}$ = Estimate of manufacturing value added in county *x* estimated with no hazard damage in the supply chain.

The 95% confidence interval for each estimated percent change was calculated using a bootstrapping procedure. This is done by estimating the impact for a random selection of observations. For this study, the process was iterated 5000 times to generate statistically significant results.

### Investment Analysis

Using the results of the simulation, an investment analysis was conducted on a USD 100 billion investment in hazard resilience. In practical terms this investment is an aggregate and would potentially arise from a combination of public and private investment. Although the reduction in losses that would result from such an investment is unknown, we can consider a selection of possibilities. For this analysis, we considered a potential 5%, 10%, 15%, 20%, 25%, and 30% reduction in losses from a USD 100 billion investment. This analysis used a 5% discount rate, a 15-year study period, and the methods for calculating net present value, internal rate of return, and payback period documented in Thomas (2017). The 5000 iterations from the bootstrapping procedure were used to develop a cumulative probability graph of the net present value.

## Results and Discussion

This study examined the effect that local hazards and hazards in the supply chain have on manufacturing GDP in the United States. It further examined the effect of varying spatiotemporal resolution in examining the effect of hazards. Three models were examined with each having a variation in the spatiotemporal unit of analysis. They include the following:

- *Model 1* refined spatial scale (county data) with broad temporal scale (annual data);
- *Model 2* broad spatial scale (state data) and broad temporal scale (annual data);
- *Model 3* broad spatial scale (state data) with refined temporal scale (quarterly data).

### Results of Regression Analysis and Hypothesis Testing

The results from the regression analysis are presented in Table 2.

**Table 2:** Results of the regression analysis

	Model 1	Model 2	Model 3	Model 4	Model 5
	County Annual	State Annual	State Quarterly	Alt County Annual	Alt State Quarterly
Value Added Lagged 1	0.8138***	0.7825***	1.1235***	0.812***	1.1137***
Value Added Lagged 2	−0.0782***	−0.1361**	−0.0071	−0.0775***	−0.0037
Value Added Lagged 3			−0.2926***		−0.2933***
Value Added Lagged 4			−0.0029		0.0076
Value Added Lagged 5			0.0055		0.0151
Value Added Lagged 6			0.0761*		0.0457**
Value Added Lagged 7			0.0335		
Value Added Lagged 8			−0.0667**		
Negative GDP Growth	−0.0796***	−0.0324***	−0.0122***	−0.0954***	−0.0124***
FAF Control	0.0244***	0.0084	0.0027	0.0298***	0.0016
Supply Chain Hazard Count Lagged 1	−0.0825***	0.0097	0.0102***		0.0118***
Supply Chain Hazard Count Lagged 2	0.0663***	0.048***	0.0041	0.0291***	
Supply Chain Hazard Count Lagged 3			0.0051		
Supply Chain Hazard Count Lagged 4			0.001		
Supply Chain Hazard Count Lagged 5			−0.0091***		−0.0078**
Supply Chain Hazard Count Lagged 6			0.0124***		
Supply Chain Hazard Count Lagged 7			0.0036		
Supply Chain Hazard Count Lagged 8			−0.0105***		−0.0086***
Value Added Lagged 1 and Hazard Count Lagged 1 Interacted	0.0009***	−0.0129**	−0.0001	0.0009***	
Value Added Lagged 2 and Hazard Count Lagged 2 Interacted	0.0001	0.0166**	0.0004		
Value Added Lagged 3 and Hazard Count Lagged 3 Interacted			0.0012		
Value Added Lagged 4 and Hazard Count Lagged 4 Interacted			−0.0003		
Value Added Lagged 5 and Hazard Count Lagged 5 Interacted			−0.0008		
Value Added Lagged 6 and Hazard Count Lagged 6 Interacted			0.0010**		0.0009**
Value Added Lagged 7 and Hazard Count Lagged 7 Interacted			−0.0005		
Value Added Lagged 8 and Hazard Count Lagged 8 Interacted			0.0006		
Hazard Count Lagged 1	−0.0100**	0.1111**	0.0012	−0.0135***	
Hazard Count Lagged 2	−0.0019	−0.1614*	−0.0061		
Hazard Count Lagged 3			−0.0118		
Hazard Count Lagged 4			0.0059		
Hazard Count Lagged 5			0.0073		
Hazard Count Lagged 6			−0.0098**		−0.0075*
Hazard Count Lagged 7			0.0058		
Hazard Count Lagged 8			−0.0063		
Hazard Damage in Supply Chain Lagged 1	−0.0013	−0.0046	−0.0015**		−0.0014*
* 95% Confidence Interval*			−*0.0029 to < 0*		−*0.0029 to < 0.0001*
Hazard Damage in Supply Chain Lagged 2	−0.0047***	−0.0013	−0.0010	−0.0032***	
* 95% Confidence Interval*	−*0.0066 to* −*0.0027*			−*0.005 to* −*0.0014*	
Hazard Damage in Supply Chain Lagged 3			0.0011		
Hazard Damage in Supply Chain Lagged 4			−0.0004		
Hazard Damage in Supply Chain Lagged 5			−0.0014*		−0.0017**
* 95% Confidence Interval*			−*0.0029 to 0.0001*		−*0.0031 to* −*0.0003*
Hazard Damage in Supply Chain Lagged 6			−0.0007		
Hazard Damage in Supply Chain Lagged 7			0.0009		
Hazard Damage in Supply Chain Lagged 8			0.0024***		0.002**
* 95% Confidence Interval*			*0.001 to 0.0039*		*0.0005 to 0.0035*
Hazard Damage Lagged 1	0.0061***	−0.0047	0.0014	0.0058***	
Hazard Damage Lagged 2	0.0016	0.0160	−0.0006		
Hazard Damage Lagged 3			0.0068		
Hazard Damage Lagged 4			−0.0012		
Hazard Damage Lagged 5			−0.0034		
Hazard Damage Lagged 6			0.0054**		0.0044**
95% Confidence Interval			0.0011 to 0.0098		0.0004 to 0.0084
Hazard Damage Lagged 7			−0.0043		
Hazard Damage Lagged 8			−0.0015		
Value Added Lagged 1 and Hazard Damage Lagged 1 Interacted	−0.0005***	0.0005	−0.0001	−0.0005***	
Value Added Lagged 2 and Hazard Damage Lagged 2 Interacted	−0.0001	−0.0019	< 0.0001		
Value Added Lagged 3 and Hazard Damage Lagged 3 Interacted			−0.0007		
Value Added Lagged 4 and Hazard Damage Lagged 4 Interacted			0.0001		
Value Added Lagged 5 and Hazard Damage Lagged 5 Interacted			0.0004		
Value Added Lagged 6 and Hazard Damage Lagged 6 Interacted			−0.0006***		−0.0005**
* 95% Confidence Interval*			−0.001 to −0.0002		−0.0009 to −0.0001
Value Added Lagged 7 and Hazard Damage Lagged 7 Interacted			0.0004		
Value Added Lagged 8 and Hazard Damage Lagged 8 Interacted			0.0001		
Indicator for No Damage Lagged 1	−0.0014	−0.0642	0.0132	−0.0100	
Indicator for No Damage Lagged 2	0.0166	−0.1077	−0.0118		
Indicator for No Damage Lagged 3			0.0368		
Indicator for No Damage Lagged 4			−0.0144		
Indicator for No Damage Lagged 5			−0.0154		
Indicator for No Damage Lagged 6			0.0113		0.006
Indicator for No Damage Lagged 7			−0.0235		
Indicator for No Damage Lagged 8			−0.0171		
Indicator for No Hazards Lagged 1	0.0144		0.0202	−0.0367	
Indicator for No Hazards Lagged 2	−0.0105		−0.0357		
Indicator for No Hazards Lagged 3			−0.0398		
Indicator for No Hazards Lagged 4			0.0538		
Indicator for No Hazards Lagged 5			0.0174		
Indicator for No Hazards Lagged 6			−0.032		−0.013
Indicator for No Hazards Lagged 7			0.0264		
Indicator for No Hazards Lagged 8			−0.0353		
Constant	3.2686***	3.4328***	1.2408***	3.0327***	1.1765***
Indicator for Quarter 2			0.0232***		0.0125***
Indicator for Quarter 3			0.0146***		0.0074*
Indicator for Quarter 4			−0.0024		−0.0042
*σ*_u	0.6129***	0.4744***	0.1682***	0.6159***	0.1516***
*σ*_e	0.1888***	0.0829***	0.037***	0.1894***	0.0373***
*ρ*	0.9133	0.9704	0.9539	0.9136	0.9429

*FAF* Freight Analysis Framework

The italics items are subcomponents

***Statistically significant at the 0.01 level; **statistically significant at the 0.05 level; *statistically significant at the 0.10 level

*Model 1* (*Annual county model*) this model examines the effect of natural hazards at the county level using annual data. As with each model, the natural hazards are lagged up to 2 years; that is, this model has both a 1-year and a 2-year lag in hazard damage. This includes a variable for local hazard damage and a variable for hazard damage in the supply chain. Therefore, there are four variables relevant to our hypotheses. Neither of the local hazard damage variables were statistically significant (see Table 2); however, the second-year lag of the supply chain damage variable was statistically significant and negative. The elasticity for this variable is −0.0047; that is, for every 1% change in supply change hazard damage, there is a −0.0047% change in manufacturing value added.

The *R*^2^ for model 1 is 0.9902 (see Table 3), which is high due to the inclusion of lagged dependent variables and most of the variation being between geographic locations. Consider, for instance, Cook County where Chicago is located compared to Loving County in Texas, which has a population under 100 people. The GDP of these two counties is vastly different and is captured by the inclusion of the lagged dependent variable. Compare this difference to the change of GDP over time, which is typically only a few percentage points of growth per year.

**Table 3:** Model details and simulation results

	Model 1	Model 2	Model 3
	County Annual	State Annual	State Quarterly
Supply Chain Damage: Lagged 2 Years	−13.9%	–	–
* Supply Chain Damage ($2016 Billions)*	−291.463	*–*	*–*
* 95% Confidence interval*	*−19.6% to −8.1%*	*–*	*–*
Supply Chain Damage: Lagged 1 Quarter	–	–	−4.4%
* Supply Chain Damage ($2016 Billions)*			*−93.2*
* 95% Confidence interval*	*–*	*–*	*−9.4% to 0.5%*
Supply Chain Damage: Lagged 5 Quarters	–	–	−2.3%
* Supply Chain Damage ($2016 Billions)*	*–*	*–*	*−49.0*
* 95% Confidence interval*	*–*	*–*	*−8.8% to 0.3%*
Observations	37,361	700	2000
*R*^2^	0.9902	0.994	0.9991

The italics items are subcomponents

*Model 2* (*Annual state model*) similar to Model 1, Model 2 has both a 1-year and a 2-year lag for local hazard damage and supply chain hazard damage. None of the four variables were statistically significant. Since none of these variables were statistically significant, no alternative model was developed. As seen in Table 3, the *R*^2^ for this model is 0.9940, which is high due to the inclusion of a lag of the dependent variable.

*Model 3* (*Quarterly state model*) this model examines the effect of natural hazards at the state level using quarterly data. This model also has lags for hazard damage for 2 years; however, since this is a quarterly model, there are eight variables for local hazard damage and eight variables for supply chain hazard damage. For the local hazard damage variable, the sixth quarter was statistically significant and positive. For the supply chain hazard damage variable the first quarter and fifth quarter variables were statistically significant and negative with elasticities of −0.0015 and −0.0014, respectively. The eighth quarter was also statistically significant, but positive. The *R*^2^ for this model is 0.9991 (see Table 3), which is high due to the inclusion of the lagged dependent variable.

*Model 4* (*Alternative annual county model*) this model is Model 1 with the hazard damage variables that were not significant being removed along with the associated count and zero indicator variables. The supply chain hazard damage variable lagged 2 years remained statistically significant as did the 1-year lag of hazard damage.

*Model 5* (*Alternative quarterly state model*) this model is an alternative version of Model 3, where the hazard damage variables that were not significant were removed along with the associated count and zero indicator variables. The first quarter lagged supply chain hazard variable and fifth quarter lagged supply chain hazard variable remained statistically significant along with the eighth quarter lagged supply chain hazard variable.

Recall that this study examined three hypotheses. The first two are regarding the effect of natural hazards on manufacturing value added and the third one is regarding varying the size of spatial units and the length of the temporal units. Below is a discuss of how the results relate to each of the hypotheses followed by a discussion of a simulation of no damage occurring as a result of hazards and an analysis of investing in hazard resilience.

*Hypothesis 1* the first hypothesis is that hazard damage occurring locally reduces manufacturing value added in subsequent years/quarters. This hypothesis was not supported by the analysis as none of the variables for hazard damage (${\mathrm{HZRD}}_{\mathrm{DMG},t-m,x}$) were statistically significant and negative. This may be due to mixed effects of hazards where hazards disrupt economic activity while at the same time demand increases due to repairs or replacement of goods along with the impact of public aid.

The literature tends to show a mix of effects from natural hazards with increasing economic growth in some sectors and decreasing growth in other sectors (Benson and Clay 2004; Loayza et al. 2012; Koks and Thissen 2016; Mohan et al. 2019). Studies frequently find that there are temporary negative effects on economic growth or that there are no lasting negative effects (Hochrainer 2009; Strobl 2011). In the short run, there are a mix of results. For instance, Albala-Bertrand (1993) showed a neutral or positive effect on economic growth (0.4% effect). On the other hand, Raddatz (2007) identified a negative effect from climatic events (2% decline in GDP per capita) and humanitarian events (4% decline in GDP per capita), however geological events were not statistically significant. Strobl (2011) identified an immediate 0.8% decline from hurricanes while Noy (2009) identified a positive 1.33% impact on GDP in the short run. Hochrainer (2009) measured a −0.5% effect on GDP after the first year of an event.

*Hypothesis 2 * the second hypothesis is that hazard damage occurring in the supply chain reduces manufacturing value added in subsequent years/quarters. This hypothesis is consistent with the evidence, as the 2-year lag of the supply chain hazard damage variable (${\mathrm{SUPCHN}}_{t-2,x}$) in Model 1 (annual county model) was statistically significant and negative. The same variable is statistically significant in Model 4, which is the alternative model for Model 1. Model 3 (quarterly state model) was also consistent with this hypothesis with the statistical significance of the one-quarter lag of the supply chain damage variable (${\mathrm{SUPCHN}}_{t-1,x}$) and the five-quarter lag (${\mathrm{SUPCHN}}_{t-5,x}$), which were both negative. The same variables remained statistically significant in Model 5, which is the alternative for Model 3. Note that all four models have statistically negative impacts in the second year. The eight-quarter lag of the supply chain variable was also statistically significant; however, it was positive, which might represent a recovery. Supply chain impacts might be delayed, as supply chains can be years long and could require time to propagate. Additionally, due to the bullwhip effect, the impact can magnify further downstream in the supply chain. A similar effect might happen going up the supply chain as well.

The lagged supply chain effects are sensible given that supply chains encompass not only spatial but temporal distance; they can be months to years long. Thomas and Kandaswamy (2015), for instance, examined the production of automobiles and aircrafts, showing that from the extraction of raw materials to the finished product can take years. We do not know the average temporal length of supply chains in the United States, but some delay in the impact would be expected, as each manufacturer maintains inventories, both material inventory and finished goods inventory; thus, even if there is a disruption, shipments might continue temporarily. Once shipments stop, the manufacturer that does not receive supplies maintains inventory that can further delay the impact of a hazard. Once inventories are depleted, production might then be interrupted in the supply chain. There is also the potential for impact due to the effect of customers—for example, they take their business elsewhere after experiencing a delay. This effect also takes time before it is realized. The results from the analysis may at first appear a bit sporadic, but both Model 1 and Model 3 show losses in the supply chain in the second year following hazards with Model 3 also showing additional losses in the first year. None of the models show local losses. In Model 3, fifth quarter losses in the supply chain might be statistically significant because at this geographical resolution, supply chains might be on average five quarters in length; however, we are uncertain of the average length and we do not know when losses occur.

*Hypothesis 3 * given that natural hazards tend to be localized in nature and given that they are largely short duration aperiodic events, broader spatiotemporal units will tend to obscure the effect on GDP, resulting in an absence of statistical significance for the effects of natural hazards. This hypothesis was consistent with the evidence in that neither the supply chain variables nor local hazard damage variables were statistically significant in Model 2, where the spatial and temporal units are broader with the geographical unit being at the state level and the temporal unit being at the annual level. The data were only available with either fine level spatial variables or fine level temporal variables, but not both. The results suggest that if data on both fine spatial units and fine temporal units were available, there might be more variables that are statistically significant. There are alternative explanations for why there is a lack of statistical significance in Model 2 (for example, misspecification); therefore, additional research is needed to confirm this hypothesis. Our results, however, suggest that spatiotemporal resolution does affect the results, as different resolutions result in different conclusions.

### Simulation

A simulation of a world with hazards without damage was run for those hazard damage variables that were statistically significant and negative. These include the 2-year lag of the supply chain hazard damage variable (${\mathrm{SUPCHN}}_{t-2,x}$) from Model 1 along with the one-quarter lag and five-quarter lag in the supply chain damage variable (${\mathrm{SUPCHN}}_{t-1,x}$ and ${\mathrm{SUPCHN}}_{t-5,x}$) in Model 3. The results are shown in Table 3. In Model 1, a 13.9% decline in manufacturing GDP is estimated to result from a 2-year lagged hazard damage in the supply chain. A 13.9% decline in manufacturing value added would amount to an approximate USD 291.5 billion in losses in 2016. For Model 3, a 4.4% decline in manufacturing value added is estimated because of a one-quarter lag in supply chain hazard damage. This would amount to a USD 93.2 billion decline in manufacturing value added in 2016. Note that the 95% confidence interval ranged from negative to positive; however, 96.7% of the bootstrap iterations used to estimate the interval were negative. An additional 2.3% decline is estimated to be the result of five-quarter lag supply chain damage, which would amount to an additional USD 49.0 billion decline. The 95% confidence interval for this estimate also ranged from negative to positive, but 95.8% of the bootstrap iterations used to estimate the interval were negative.

### Investment Analysis

An investment analysis was conducted using Model 1 and Model 3 results. In Model 1, the second-year losses are reduced by six different levels, each at 5% increments, as previously discussed. In Model 3, the first-quarter losses and fifth-quarter losses were reduced by the same six levels, varying at 5% increments. As seen in Table 4, 10 of the 12 investment analyses were found to be economical with the net present value being positive and the internal rate of return exceeding the discount rate (that is, 5%). Only the two 5% reduction levels were found to be uneconomical. Moreover, the results suggest that if a USD 100 billion investment in hazard resilience has a 10% or greater reduction in losses, then it is an economical investment.

**Table 4:** Net present value and internal rate of return for a USD 100 billion investment in hazard resilience

Reduction in Losses (%)	Net Present Value $Billions 2016	Internal Rate of Return (%)	Payback Period (Years)
Model 1			
5	51.3	11.9	7
10	202.5	28.5	4
15	353.8	43.5	3
20	505.1	58.2	2
25	656.3	72.8	2
30	807.6	87.4	2
Model 3			
5	−26.2	0.8	15
10	47.6	11.4	8
15	121.4	19.9	5
20	195.1	27.7	4
25	268.9	35.2	3
30	342.7	42.4	3

**Fig. 2:**
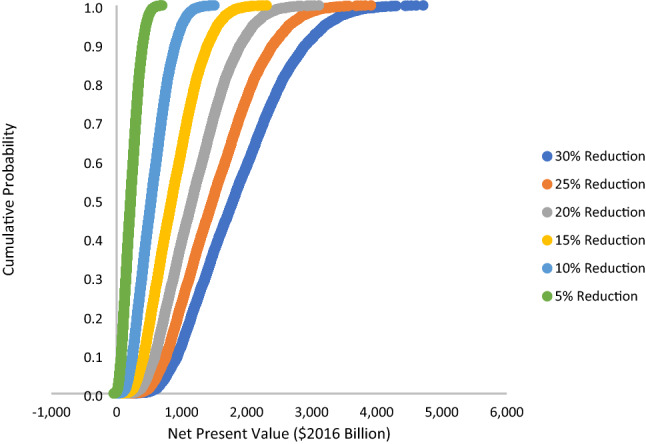
Net present value of a USD 100 billion investment in hazard resilience: Model 1 (annual county model) cumulative probability by level of loss reduction

**Fig. 3:**
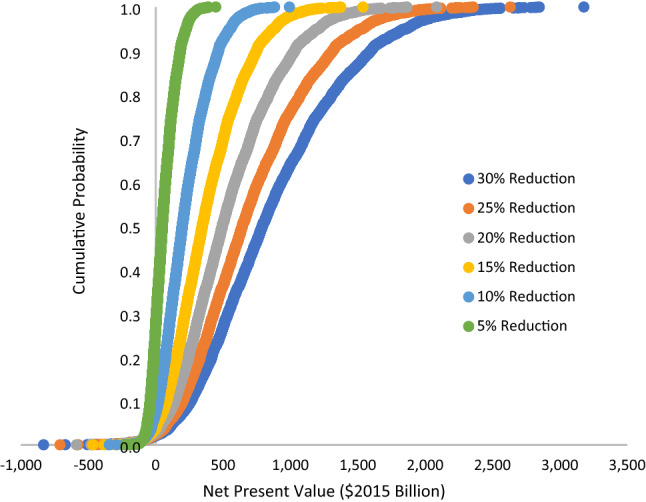
Net present value of a USD 100 billion investment in hazard resilience: Model 3 (state quarterly model) cumulative probability by level of loss reduction

A cumulative probability graph was generated for each model at each level of reduction using the iterations from the bootstrapping procedure (see Figs. 2, 3). Each iteration from the bootstrapping procedure was used to estimate the net present value of investing in resilience with the potential for loss reduction at the six reduction levels (that is, between 5 and 30%, varied at 5% intervals). At the 5% reduction level, 73% of the iterations for Model 1 were economical with positive net present value. At the 10%, 15%, 20%, 25%, and 30% loss reduction levels 92.7%, 96.1%, 97.2%, 97.6%, and 97.9% of the iterations were economical, as illustrated in Fig. 2. For Model 3, at the 5% reduction level, 99.6% of the iterations were economical and for the remaining reduction levels 100% were economical, as illustrated in Fig. 3. Moreover, 73% to 100% of the iterations were economical. For all levels of reduction examined from both models, 96.2% of the iterations were economical.

## Conclusion

This study examined three hypotheses with two of them being supported by the analysis. The variable for local hazard damage was not statistically significant in any of the models, suggesting that local hazard damage does not decrease local manufacturing value added. This may be due to mixed effects of hazards where some demand increases due to repairs or replacement of goods. The second-year lag of hazard damage in the supply chain in Model 1 along with the one-quarter lag and fifth-quarter lag in Model 3 of the same variable were statistically significant, showing that hazard damage in the supply chain has a negative effect on manufacturing locally. Finally, the lack of statistical significance of either local or supply chain effects in Model 2 suggests that spatiotemporal variations in the variables influence the analysis; however, other functional forms (that is, variations in the model) may result in statistical significance for the variables of interest. Although our results suggest that the spatiotemporal resolution affects results, additional research is needed to further confirm this hypothesis. Natural hazards tend to be local in nature and are short duration aperiodic events. The effects of one or more events might be lost in the noise of broader spatiotemporal variables especially if hazards are more likely to occur during certain seasons and/or at certain locations, as a high-risk combination in time and space would be combined with low-risk combinations. Additionally, there might be some firms that experience a positive effect from a hazard while others experience a negative effect. Broader spatiotemporal units combine many of these mixed effects together and may in aggregate dampen otherwise pronounced impacts at given points in the supply chain.

A simulation and investment analysis of investing in hazard resilience were also conducted. To the extent possible, this study aimed to measure the negative effects of hazards or the losses rather than the net effect. An increase in demand in another industry is little consolation for those who did not benefit and whose businesses are disrupted and experience losses. The simulation from Model 1 shows a 13.9% decline in manufacturing value added results from natural hazards. Model 3 shows a 4.4% decline in quarter one and a 2.3% decline in quarter five. An investment analysis was conducted using a 15-year study period and a 5% discount rate. The results show that a USD 100 billion investment in hazard resilience is economical if it reduces the simulated losses by 10% or more. A bootstrapping procedure resulted in 73% to 100% of the iterations being economical, depending on the model and level of loss reduction.

We examined the effect of all hazards together. Future research might examine the comparative impacts across natural hazard types (that is, acute or chronic). Different hazard types are likely to have varying effects, in part due to learning through experience by firms along the respective supply chains. A better understanding of these differences will advance our understanding of how hazards affect GDP and how resilience planning may help reduce the indirect and ancillary effects. The results indicate that there are potentially significant losses in the supply chain due to hazards and further research is warranted to confirm or disprove these findings. Our results suggest that in conducting further research, it is important to consider the spatiotemporal resolution of the data being used, as lower resolution might obscure the results. Future research should also consider that common approaches to examining supply chain losses (for example, computable general equilibrium modeling or input-output analysis) may not capture the losses demonstrated in this study, as they often represent an equilibrium or change in demand and do not include the effect of goods failing to arrive on time at the next point in the supply chain. These methods are indispensable for community planning but may not estimate all supply chain losses at all points in time following a hazard.
